# HPV18 Utilizes Two Alternative Branch Sites for E6*I Splicing to Produce E7 Protein

**DOI:** 10.1007/s12250-019-00098-0

**Published:** 2019-04-03

**Authors:** Ayslan Castro Brant, Vladimir Majerciak, Miguel Angelo Martins Moreira, Zhi-Ming Zheng

**Affiliations:** 1 Tumor Virus RNA Biology Section, RNA Biology Laboratory, Center for Cancer Research, National Cancer Institute, NIH, Frederick, MD 21702 USA; 2 Genetics Post-Graduation Program, Rio de Janeiro Federal University, Rio de Janeiro, Brazil; 3 Genetics Program, Nacional Cancer Institute, INCA, Rio de Janeiro, 20231-050 Brazil

**Keywords:** Human papillomavirus 18 (HPV18), HPV splicing, Branch point, E6, E7, E6 intron, HPV oncogenes

## Abstract

Human papillomavirus 18 (HPV18) E6 and E7 oncogenes are transcribed as a single bicistronic E6E7 pre-mRNA. The E6 ORF region in the bicistronic E6E7 pre-mRNA contains an intron. Splicing of this intron disrupts the E6 ORF integrity and produces a spliced E6*I RNA for efficient E7 translation. Here we report that the E6 intron has two overlapped branch point sequences (BPS) upstream of its 3′ splice site, with an identical heptamer AACUAAC, for E6*I splicing. One heptamer has a branch site adenosine (underlined) at nt 384 and the other at nt 388. E6*I splicing efficiency correlates to the expression level of E6 and E7 proteins and depends on the selection of which branch site. In general, E6*I splicing prefers the 3′ss-proximal branch site at nt 388 over the distal branch site at nt 384. Inactivation of the nt 388 branch site was found to activate a cryptic acceptor site at nt 636 for aberrant RNA splicing. Together, these data suggest that HPV18 modulates its production ratio of E6 and E7 proteins by alternative selection of the two mapped branch sites for the E6*I splicing, which could be beneficial in its productive or oncogenic infection according to the host cell environment.

**Electronic supplementary material:**

The online version of this article (10.1007/s12250-019-00098-0) contains supplementary material, which is available to authorized users.

## Introduction

Human papillomaviruses (HPV) are small, non-enveloped DNA viruses and contain a double-stranded DNA genome ~8 kb in size. The HPV genome encodes eight open-reading frames (ORF), of which six (E1, E2, E4, E5, E6 and E7) are encoded from the genome early region and two (L1 and L2) are encoded from the late region (Zheng and Baker 2006). More than 200 different genotypes of HPV have been identified (Ranjeva *et al.*^2017^). The mucosotropic HPV can be clinically classified as low-risk HPV (LR-HPV), producing benign warts, and high-risk HPV (HR-HPV), whose infections lead to development of cervical, genital and oropharyngeal cancers (Walboomers *et al.*^1999^; de Villiers 2013). Among HR-HPV, HPV16 and HPV18 are the two most frequent genotypes detected in cervical cancer (zur Hausen 2002).

HR-HPV E6 and E7 are two viral oncoproteins that respectively, induce protein degradation of tumor suppressors p53 and pRb (Vousden 1994). Unlike LR-HPV from which E6 and E7 are transcribed from separate promoters (Zheng 2010), HR-HPV E6 and E7 are transcribed from the same promoter as a single bicistronic E6E7 pre-mRNA (Zheng and Baker 2006; Wang *et al.*^2011^). The E6 ORF in this bicistronic pre-mRNA contains an intron (also called the E6 intron) which is subject to alternative splicing. Escaping of the E6 intron from RNA splicing leads to remain the E6 ORF integrity and is necessary for E6 protein translation. However, splicing of the E6 intron during HR-HPV infection is highly efficient and is required for E7 protein translation (Zheng *et al.*^2004^; Zheng and Baker 2006; Tang *et al.*^2006^). Majority of the spliced product with a disrupted E6 ORF is the E6*I which serves as an E7 mRNA for E7 protein translation (Tang *et al.*^2006^). Currently, it remains unclear how the E6 intron splicing is regulated for E7 expression, and how the E6 intron could escape from RNA splicing to express E6 protein during HR-HPV infection are under active investigation. HPV18 E6 and E7 are transcribed from two alternative early promoters, P102/105 and P55, upstream of the E6 ORF as a single bicistronic primary E6E7 transcript (Wang *et al.*^2011^, ^2016^). The E6 intron in the HPV18 E6E7 bicistronic pre-mRNA transcripts has one splice donor site (5′ splice site or 5′ss) at nt 233 and two alternative splice acceptor sites (3′ splice site or 3′ss), one at nt 416 and the other at nt 791. The alternative usage of these two 3′ splice sites results in two spliced mRNAs: the major E6*I with a 233^416 splicing junction to serve as an E7 mRNA and the minor E6*X with a 233^791 splicing junction to encode an E6^E7 fusion protein (Zheng *et al.*^2004^; Ajiro and Zheng 2015a).

In eukaryotes, introns are removed from pre-mRNAs in the spliceosome. The spliceosome complex are composed of proteins and small nuclear RNAs (snRNAs) (Will and Lührmann 2011; Shi 2017). RNA splicing requires four intronic elements: (i) a 5′ss with a GU dinucleotide at the intron 5′ end, (ii) a 3′ss with an AG dinucleotide at the intron 3′ end, (iii) a branch point sequence (BPS), and (iv) a polypyrimidine tract (PPT) between the 3′ss and BPS. Usually, the BPS, located 15–40 nucleotides upstream of the 3′ss, is comprised of 5 (YUNAY) (Gao *et al*. 2008; Mercer *et al.*^2015^) or 7 (YNYURAC) (Zhuang *et al.*^1989^; Kol *et al.*^2005^) nucleotides with one adenosine (underlined) as the branch site. During pre-mRNA splicing, the 5′ss is first recognized by U1 snRNA, the BPS by U2 snRNA, and the 3′ss by U2AF. Following these recognitions and crosstalking, the intron is excised by two trans-esterification reactions. In the first reaction, the hydroxyl ·OH radical of the branch site adenosine attacks the phosphodiester bond at the intron 5′ end G at the 5′ss, cleaving the 5′-exon from the intron. At this stage, the intron forms a lariat-intermediate structure with a 5′–2′ phosphodiester bond between the 5′ss guanosine and the branch site adenosine. In the second reaction, the ·OH radical of the released 5′-exon attacks the phosphodiester bond of the intron 3′ss exon in the lariat intermediate, leading the cleavage of the intron 3′ss from the 3′-exon and linking the 5′-exon to the 3′-exon. The intron is then released as a lariat conformation and degraded by de-branching enzymes (Will and Lührmann 2011; Shi 2017).

Although BPS is crucial for mRNA splicing in eukaryotes, mapping a BPS for individual intron splicing and understanding its regulation remain challenge. Various approaches in combination with computational bioinformatics have been attempted to enrich genome-wide circular forms of the lariats or lariat intermediates for BPS mapping (Gao *et al.*^2008^; Taggart *et al.*^2012^; Mercer *et al.*^2015^) with few successfully validated cases of a few selected genes. A recent report indicated that almost all human introns contain multiple branchpoints and exhibit tissue-specific branchpoint usage (Pineda and Bradley 2018). Using a lariat RT-PCR technology in combination of TA cloning-sequencing, our lab has mapped the BPS for RNA splicing of the BRAF intron 8 (Ajiro and Zheng 2015b), the BPV-1 late RNA intron 1 (Zheng *et al.*^2000^) and the HPV16 E6 intron (Ajiro *et al.*^2012^). In this report, we discovered that the HPV18 E6 intron contains two alternative branch sites at nt 384 and 388, but preferentially uses a 3′ss-proximal nt 388 adenosine as a branch site for E6E7 pre-mRNA splicing. Introduction of mutations at nt 388 and 384 blocked selection of the nt 416 3′ss for E6*I splicing, but activated the usage of a cryptic acceptor splicing site at nt 636, leading to a novel splicing isoform from nt 233^636.

## Materials and Methods

### Computational Analysis for BPS Detection

Computational analysis of the 3′ end 100-bp region (from position 316 to 415) of HPV18 E6 intron was carried out to identify a potential BPS with Human Splice Finder 3.1 (http://www.umd.be/HSF3/HSF.shtml) and default parameters. A BPS candidate is a seven-nucleotide motif (heptamer) with a consensus value (CV) calculated by its nucleotide similarity with the consensus sequence of the mammalian BPS (Desmet *et al.*^2009^).

### Plasmids

A HPV18 E6 and E7 region (nt 103–967) with mutations at the BPS candidate motifs was amplified from the plasmid pZMZ84 originated from HeLa HPV18 genome by overlapping PCR with the corresponding primer sets (Supplementary Table S1) and inserted into a pcDNA3 vector (Invitrogen) between *Hin*d III and *Not* I sites. The constructed plasmid pAYS2 had a single A384G mutation (mt-1); pAYS7 had a single A388G mutation (mt-2); and pAYS3 had both A384G and A388G mutations (mt-3). Additional constructs based on pFLAG-CMV-5.1 (Sigma-Aldrich) were used for *in vivo* RNA splicing and translation of Flag-tagged E6 and E7 proteins with the Flag-tag at the N-terminal E6 and at the C-terminal E7, respectively. All plasmids containing HPV18 E6 and E7 regions (nt 105-904) were constructed by overlapping PCR with the corresponding primer sets. The resulted plasmid pAYS8 had both wild type E6 and E7 ORFs (wt); pAYS9 had the A384G mutation (mt-1); pAYS11 had A388G mutation (mt-2); and pAYS10 had both A384G and A388G mutations (mt-3).

### *In vitro* RNA Transcription and Splicing

To generate the DNA templates for *in vitro* transcription, the HPV18 E6 ORF region (nts 123 to 500) was PCR amplified using oST247 and oSB70 (Supplementary Table S1) to introduce T7 promoter (5′ end) and U1 snRNP-binding site (3′) from plasmid pMA77 (Ajiro *et al.*2016b) containing wt and pAYS2, pAYS3 or pAYS7, containing mutated BPS as described above. One µg of PCR templates were then used for *in vitro* transcription using Riboprobe System-T7 (Promega) in a 40-µL reaction containing 1× transcription buffer, 10 mmol/L DTT, 40 U RNase inhibitor, 0.5 mmol/L m7G(5´)ppp(5´)G cap analogue (New England Biolabs), 0.5 mmol/L of each rNTP (rATP, rUTP, rCTP, rGTP) and 60 U T7 RNA polymerase. The reactions were incubated at 37 °C for 2 h followed by DNA template removal by 30 min treatment with RNase-free DNase I (Promega). Resulting RNA was purified by ultrapure phenol:chloroform:isoamyl alcohol (25:24:1, v/v, Thermo Fisher Scientific) extraction, precipitated, and dissolved in DEPC-treated water. Alternatively, the *in vitro* transcribed RNA was radiolabeled by addition of 60 µCi [α-^32^P]-rGTP (Perkin Elmer) into the transcription reaction containing reduced level (0.05 mmol/L) of cold rGTP. The radiolabeled RNA was gel-purified on a 6% denaturing urea-PAGE gel (National Diagnostics). *In vitro* splicing was carried out with 100 ng of non-labeled or 4 ng of ^32^P-labeled of HPV18 E6 pre-mRNA in the presence of HeLa nuclear extract as previously reported (Zheng and Baker 2000). After 2 h incubation the spliced products were purified by phenol:chloroform:isoamyl alcohol extraction, precipitated and dissolved in DEPC-treated water. The spliced products generated from unlabeled RNA was used in lariat RT-PCR. The splicing products of radiolabeled RNA were separated on a 6% denaturing urea-PAGE gel (National Diagnostics), transferred to a filter paper and dried. The signals were captured by Amersham Typhoon 5 laser scanner (GE Healthcare) and signal intensity of each spliced product was determined using ImageQuant software (GE Healthcare). RNA splicing efficiency was calculated as described (Zheng and Baker 2000).

### RNase R Treatment and Lariat RT-PCR

To remove the linear part of lariat RNA intermediates, *in vitro* spliced products from cold E6 pre-mRNA (50 ng) or from total RNA extracted from HeLa cells (10 µg), were treated with 40U of RNase R (Epicentre) (Suzuki *et al.*^2006^) in a 50-µL reaction at 37 °C for 2 h. Treated RNA was extracted with phenol:chloroform:isoamyl alcohol, precipitated, dried, and dissolved in DEPC-treated water. RNase R- digested products were used to identify the 5′–2′ bond in the circular lariat structure required for the first step RNA splicing of the E6 intron by lariat RT-PCR (Ajiro and Zheng 2015b): first, circular RNA was reversely transcribed with 200 U of SuperScript II reverse transcriptase (Thermo Fisher Scientific) with a primer oAYS12 (R) and the cDNA was subsequently PCR-amplified with a primer pair of oAYS12 (R) and oAYS13 (F1) primers followed by a semi-nested PCR with another primer pair of oAYS12 (R) and oAYS8 (F2) primers (Supplementary Table S1). Amplified products were excised from agarose gels, cloned into pCRII TOPO vector using TOPO TA cloning kit (Thermo Fisher Scientific) and sequenced.

### Transfections, RT-PCR and Western Blotting

Human osteosarcoma U2OS cells and human embryonic kidney HEK293T cells were cultivated in McCoy′s 5a or in DMEM media, respectively, supplemented with 1 × Penicillin–Streptomycin-Glutamine (Thermo Fisher Scientific) and 10% fetal bovine serum (GE Healthcare). Approximately 3 × 10^6^ of each cells were plated in a separate 60-mm dish and transfected with 2 µg of Flag-tagged plasmid pAYS8, pAYS9, pAYS10 or pAYS11 with LipoD293 (SignaGen Laboratories) or an empty vector (pFLAG-CMV-5.1) as a control. Each dish was treated at 24 h after transfection with proteasome inhibitor MG132 (Sigma-Aldrich) (Ajiro and Zheng 2015a) at a final concentration of 10 µmol/L for additional six hours before sample collection. Total RNA was isolated with TRIzol (Roche) and total protein was extracted by addition of 250 µL (to U2OS cells) or 500 µL (to HEK293T cells) of 2.5 × SDS protein gel loading solution with 10% β-mercaptoethanol.

RNA from transfected cells was treated with TURBO DNA-free kit (Thermo Fisher Scientific) to remove plasmid DNA contamination, and reverse transcription (RT) was carried out with Moloney murine leukemia virus reverse transcriptase (M-MLV RT, Thermo Fisher Scientific) and random hexamer primers. Following RT, PCR was carried out with AmpliTaq (Thermo Fisher Scientific) and a primer pair of oZMZ252 plus oZMZ253 (Supplementary Table S1) to amplify HPV18 E6E7, and a primer pair of oZMZ269 plus oZMZ270 (Supplementary Table S1) to amplify GAPDH (as a loading control). The efficiency of splicing was determined based on amount of individual splicing products separated in an ethidium bromide-containing agarose gel and their signal intensities measured by Image Lab software (Bio-Rad).

Total protein samples were separated on NuPAGE 4%–12% Bis–Tris gels (Thermo Fisher Scientific), transferred to a nitrocellulose membrane and subsequently blotted with the following primary antibodies: rabbit anti-FLAG polyclonal antibody (F7425, Sigma-Aldrich), goat anti-E7 HPV18 polyclonal antibody (SC-1590, Santa Cruz Biotechnology) or mouse anti-β-tubulin monoclonal antibody (T5201, Sigma-Aldrich). Subsequently, the membrane was blotted with a secondary antibody (anti-rabbit, anti-goat or anti-mouse) conjugated with horseradish peroxidase (Sigma-Aldrich). The immunoreactive proteins were detected with enhanced chemiluminescence using SuperSignal West Pico PLUS Chemiluminescent Substrate (Thermo Fisher Scientific). The signal was captured by ChemiDoc Touch imaging system (Bio-Rad) or on a X-ray film. The membrane was stripped with Restore Plus Western Blot Stripping Buffer (Thermo Fisher Scientific) and reblotted with another primary antibody.

## Results

### BPS Prediction by Computational Analysis

**Fig. 1:**
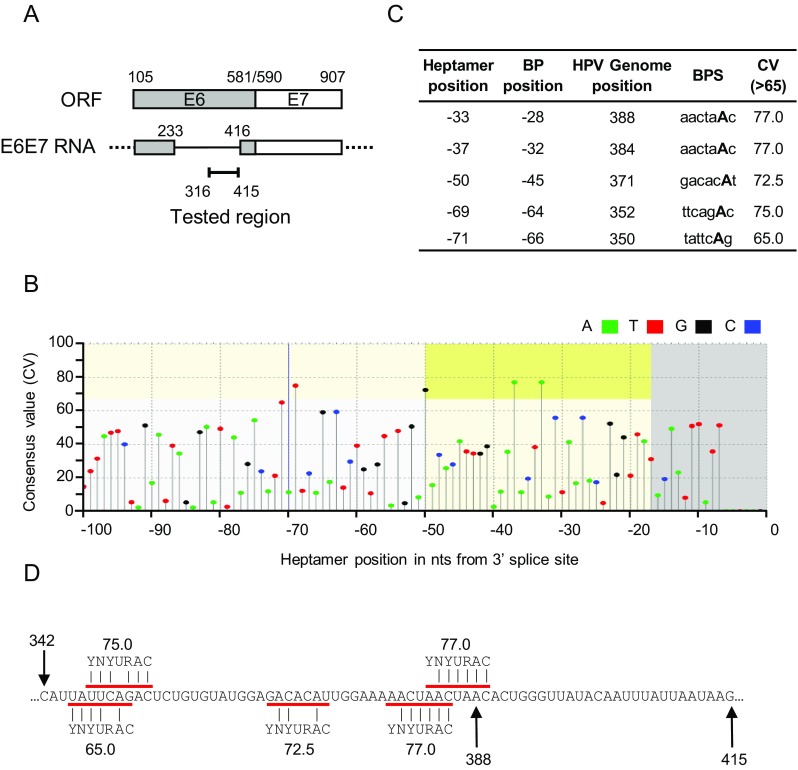
Computational analysis of HPV18 E6 intron for potential branch point sequence (BPS). **A** Diagram of the E6 (grey box) and E7 (white box) ORFs in the HPV18 genome and the structure of transcribed bicistronic E6E7 mRNA. The numbers represent nucleotide positions in the HPV18 reference genome (GenBank number: X05015). Dash lines on each side of the E6E7 RNA are the untranslated regions. Below is the 100 nt test region (from genomic position 316 to 415) representing the 3′ end of the E6 intron used for prediction of potential BPS by Human Splicing Finder 3.1 software (http://www.umd.be/HSF3/index.html). **B** Graphical representation of computationally predicted BPS showing their score and distance of the first nucleotide (colored dots) of a putative heptameric BPS from the splice acceptor site. Heptameric BPS located in the yellow area appears as a higher probability of the putative BPS utilized for splicing of the E6 intron. **C** Positions from the 3′ ss and in the HPV18 genome of five putative BPS sequences with a consensus value (CV) higher than 65 from (B). A bold A at the sixth base of the heptameric sequence indicates a putative branch site adenosine in the putative BPS. **D** Sequence comparison between each putative BPS and mammalian consensus BPS (YNYURAC).

Given the BPS posits usually at 15–40 nts upstream of a 3′ss, we screened a 100-bp long region to the HPV18 E6 intron 3′ end from nt 316 to nt 415 (Fig. 1A) for a consensus value (CV) of each nucleotide against the mammalian BPS sequence (YNYURAC) by using the Human Splice Finder 3.1 (http://www.umd.be/HSF3/index.html). The obtained CVs for each nucleotide are shown in Fig. 1B. From this analysis, we found that five heptamer sequences with an A at the 6th position exhibited a CV above 65 (ranging from 65 to 77) that resemble to a BPS motif (Fig. 1C and 1D). Interestingly, two heptamers, with the highest scores, are overlapped each other and contain an adenosine at the 6th position at nt 384 for one heptamer or at nt 388 for the other heptamer (Fig. 1D). Both have the same sequence composition with six of seven nucleotides matching to the mammalian BPS consensus sequence YNYURAC (Fig. 1D).

### Lariat RT-PCR for BPS Identification

**Fig. 2:**
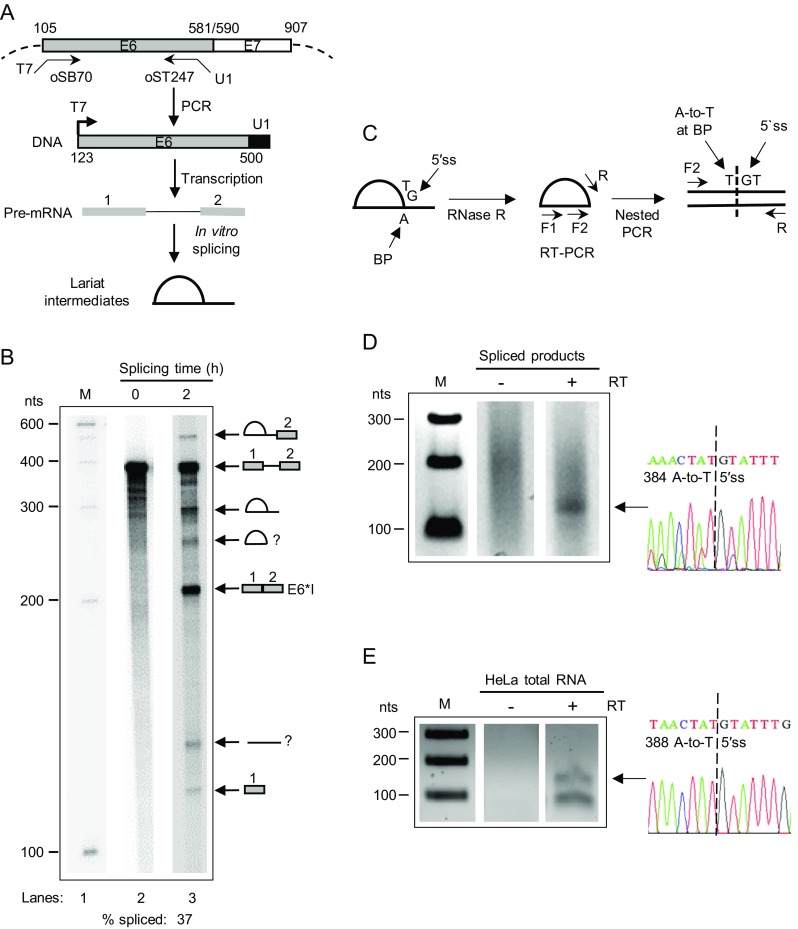
Mapping of HPV18 E6 intron BPS by lariat RT-PCR. **A** Schematic strategy of preparing HPV18 E6 RNA transcripts for *in vitro* RNA splicing assays. The selected E6 region (grey box) was amplified by PCR from an E6E7 minigene plasmid pMA77 (Ajiro *et al*. 2016b) and the generated DNA template for *in vitro* transcription contains a T7 promoter (arrow) upstream and a U1-binding site downstream (black box) of the E6 ORF region. The transcribed pre-mRNA was used for *in vitro* splicing reaction using HeLa nuclear extract to generate the lariat intermediates. **B** Splicing efficiency of E6 pre-mRNA. An *in vitro* RNA splicing assay was carried out with 4 ng of [α-^32^P] GTP-labeled HPV18 E6 pre-mRNA in the presence of HeLa nuclear extract at 30 °C for 2 h. The RNA at 0 h of the splicing reaction served as a control. The corresponding splicing products were resolved in a 6% denaturing PAGE-gel and are diagramed on the right of the splicing gel. The splicing efficiency of E6 pre-mRNA is expressed as a percentage of total spliced products/total pre-mRNA input and shown below the splicing gel. **C** The strategy for lariat RT-PCR. The lariat-containing RNA was treated with RNase R to remove its linear portion and then served as a template for RT-PCR first using a gene-specific-reverse (R) primer and a forward (F1) primer and then nested PCR using the R primer and the F2 primer. The resulting PCR product contains the junction between the donor splice site (5′ss) and the branch site (BS) often with an A-to-T substitution. **D** and **E** The lariat RT-PCR product amplified from *in vitro* spliced HPV18 E6 pre-mRNA (**D**) or total RNA from HeLa cells (**E**). The reactions without RT (–) were used as a negative control. The obtained products were gel-purified, cloned and sequenced. On the right are the sequence chromatographs showing the mapped branch site junction, with an A-to-T substitution, to 5′ss.

To confirm the above prediction, we first transcribed *in vitro* the E6 region (nt 123–500) in the HPV18 genome from a chimeric T7-HPV18 E6 DNA template generated by PCR. A U1-binding site was attached to the E6 pre-mRNA 3′ end to serve as a splicing enhancer (Ajiro *et al.*^2012^). The transcribed E6 pre-mRNA containing the E6 intron was used for *in vitro* splicing reaction in the presence of HeLa nuclear extract (Fig. 2A). Under this condition, the E6 pre-mRNA was spliced efficiently with production of the fully spliced products and spliced intermediates (Fig. 2B). Subsequently, these spliced products were used to detect the splicing lariats which are formed by the intron 5′ GU linking to the branch site adenosine in a BPS via 5′–2′direction from the first step of RNA splicing. The circular lariat and its intermediates were detected by lariat RT-PCR with two pairs of primers in the opposite directions, first by the primer pair F1 and R and then by the nested primer pair F2 and R (Fig. 2C). The amplified RT-PCR product in size of approximate 130-bp derived from the circular lariats was gel-purified, cloned and sequenced. We demonstrated that the splicing lariats were formed in *in vitro* RNA splicing by linkage of the intron 5′ss G at nt 234 to an adenosine at nt 384 (Fig. 2D). Subsequently, total RNA from HPV18-positive HeLa cells was analyzed by this lariat RT-PCR strategy described above and got an amplified product with a similar size of approximate 130 bps. Gel-purification, cloning and sequencing analysis showed that the amplified lariat product from HeLa cells was branched from an adenosine at nt 388 (Fig. 2E) which was at a different position from the mapped branch site at nt 384 from the *in vitro* spliced lariat intermediates. Nevertheless in both cases, an A-to-T transition at the mapped branch site (also called branch point) was observed as expected (Vogel *et al.*^1997^; Zheng *et al.*^2000^; Conklin *et al.*^2005^; Ajiro and Zheng 2015b).

### Verification of the Mapped BPS by Point Mutation and *In vitro* RNA Splicing

**Fig. 3:**
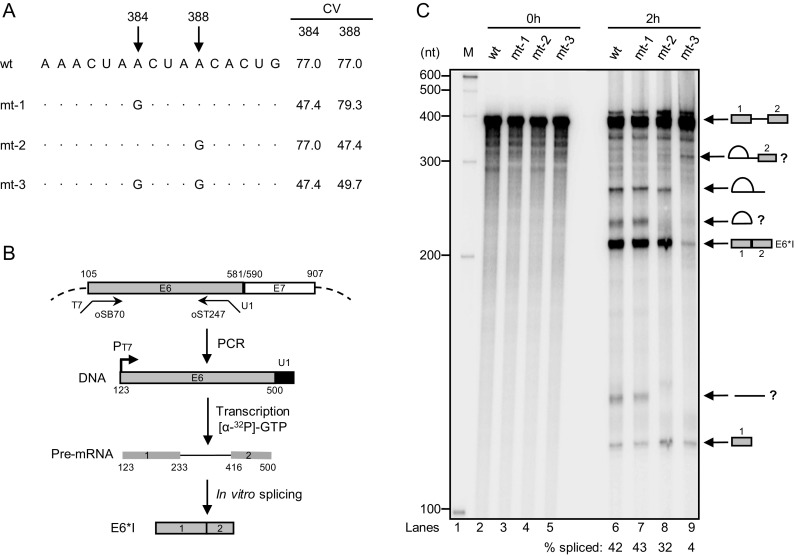
Mutational analysis of the mapped branch site in the HPV18 E6 intron. **A** Introduction of single or double A-to-G mutations into the mapped branch site adenosine 384 or/and 388. Dots indicate the identical nucleotides to the wild type (wt) sequence. On the right side is a consensus value (CV) of each BPS before and after mutation and was calculated by Human Splicing Finder 3.1. **B** A strategic flow-chat in preparation of E6 pre-mRNA containing a wt or mt BPS (**A**) for *in vitro* splicing studies. The E6 ORF region (grey box) with or without a branch site mutation in the mapped BPS was amplified by PCR from individual E6E7 minigene plasmids and the PCR DNA template for *in vitro* transcription has a T7 promoter (arrow) upstream and a U1-binding site downstream (black box) of the E6 ORF region. The transcribed pre-mRNA labeled with [α-^32^P]-GTP was used in *in vitro* splicing assay in the presence of HeLa nuclear extract to produce a fully spliced E6*I product. **C** Effect of branch site mutations in the mapped BPS on E6 intron splicing. *In vitro* splicing of an E6 RNA with a wt or mt BS was carried out in the presence of HeLa nuclear extract for 2 h. The RNA at 0 h of the splicing reaction served as a control. The corresponding splicing products are diagramed on the right. The splicing efficiency of each E6 pre-mRNA is expressed as a percentage of total spliced products/total pre-mRNA input and shown below the splicing gel.

To analyze the effect of point mutations at nt 384 and nt 388 on splicing of the E6 intron, constructs containing a wild type, single or double (A-to-G) mutations of the mapped branch sites (Fig. 3A) were used for an *in vitro* transcription and RNA splicing (Fig. 3B). We noticed that introduction of A-to-G mutation at these two positions led to reduce the consensus value of each mapped BPS (Fig. 3A). As shown in Fig. 3C, analyses of spliced products in a 6% urea PAGE gel indicated that both pre-mRNAs of wt and mt-1 with an A-to-G mutation at nt 384 exhibited a similar *in vitro* splicing efficiency (42% and 43%) at a 2-hour splicing reaction (compare lane 6 to lane 7). On the other hand, splicing efficiency of the mt-2 pre-mRNA with an A-to-G mutation at nt 388 displayed 10% lower splicing efficiency than that of wt pre-mRNA, accompanied by less amount of lariat products and accumulation of the spliced exon 1 (Fig. 3C, compare lane 8 to lane 6). Interestingly, introduction of the A-to-G mutations to both nt 384 and nt 388 in mt-3 pre-mRNA was found to be detrimental for the mt-3 splicing, leading the splicing efficiency of mt-3 pre-mRNA dropping to only 4% without detectable lariats (Fig. 3C, compare lane 9 to lane 6). These data clearly indicate that the nt 384 and nt 388 in HPV18 E6 intron act as two alternative branch points for E6 intron splicing *in vitro*.

### Function of the Mapped Branch Points at nt 384 and nt 388 in HPV18 E6E7 RNA Splicing and Oncoprotein Production in HEK293T and U2OS Cells

**Fig. 4:**
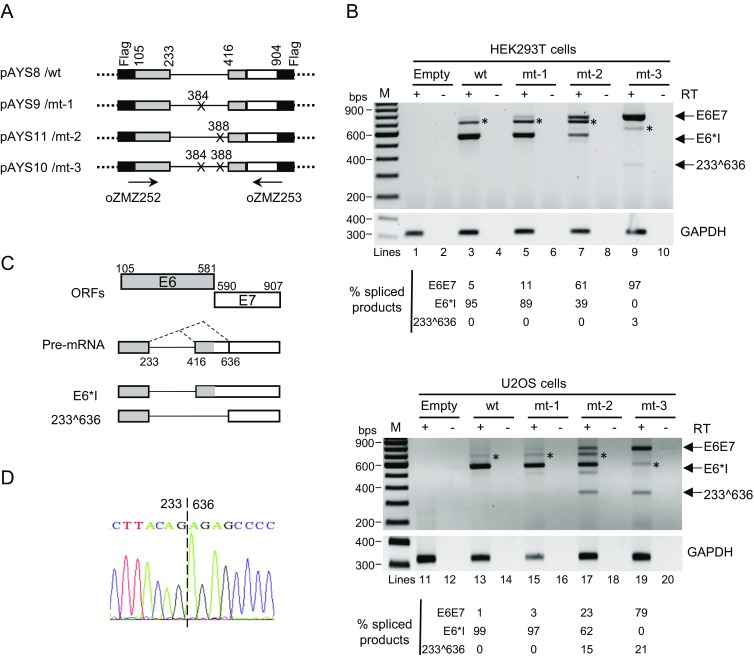
Role of the mapped branch site usage in splicing of HPV18 E6E7 RNA in HEK293T and U2OS cells. **A** Construction of Flag (black box) fused E6 (grey box) and E7 (white box) minigene expression vectors. Thin line represents the HPV18 E6 intron with a wt or mt branch site (X, A-to-G) at nt 384 (mt-1) or nt 388 (mt-2) or both (mt-3) (also see Fig. 3A). **B** Splicing profiles of HPV18 E6E7 pre-mRNA in HEK293T and U2OS cells transfected with individual E6E7 minigene plasmids as shown in (A). The cells transfected with an empty vector were used as a negative control. Total RNA isolated from the transfected cells was analyzed by RT-PCR with the indicated primer pair of oZMZ252 and oZMZ253 (A). The identity of each band corresponding to various spliced products is shown at right, and the quantification (in percentage) of spliced RNA is shown below the gel. The asterisk (*) in transfected HEK293T and U2OS cells indicates a heteroduplex band derived from two RT-PCR products (Ajiro *et al.*2016b). **C** Summary of all spliced products of E6*I and/or 233^636 detected in HEK293T and U2OS cells transfected with an E6E7 minigene. **D** The chromatograph obtained by sequencing of a novel splicing product due to branch site mutation showing the splicing of 5′ss at nt 233 to a new, cryptic splice acceptor site at nt 636 (233^636).

**Fig. 5:**
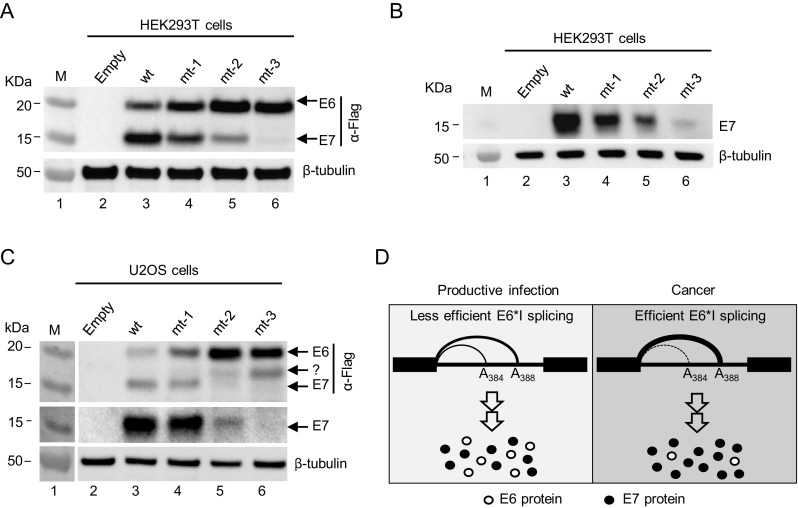
Role of the mapped branch site usage in splicing of HPV18 E6E7 RNA and expression of E6 and E7 proteins in HEK293T and U2OS cells. (**A** and **B**) Total protein extracted from HEK293T cells transfected with a wt or mutant HPV18 E6E7 minigene vector was blotted for expression of Flag-tagged E6 (18.3 kDa) and/or E7 (13.6 kDa) by an anti-Flag antibody (**A**) or anti-HPV18 E7 antibody (**B**). **C** Total protein extracted from U2OS cells transfected as described above was blotted for the expression of Flag-tagged E6 and E7 by the anti-Flag antibody and then by anti-HPV18 E7 antibody (E7, middle panel). Cellular β-tubulin served as a sample loading control in all three panels of A, B and C. **D** Regulated branch site selection in HPV18 E6 and E7 splicing contributes to E6 and E7 protein production and oncogenesis. This illustration hypothesizes that selection of two alternative branch sites for splicing of the E6 intron correlates to the efficiency of E6*I splicing and regulates the production of E6 and E7 proteins. Selection of the nt 384 branch site in productive HPV18 infection leads to less efficient splicing and retention of the E6 intron and thereby makes the E6 ORF intact to express E6 protein, although the nt 388 branch site is proportionally preferred over the nt 384 branch site. In the HPV18-induced cancer, high efficiency of E6*I splicing by overwhelming selection of the 388 branch site provides more E6*I RNAs for translation of E7 to maintain the cancer cell growth.

Given that both branch sites at nt 384 and nt 388 are essential for *in vitro* splicing, we further analyzed their effects on HPV18 E6E7 mRNA splicing *in vivo* and production of E6 and E7 oncoproteins. For this purpose, we constructed various E6E7 expression vectors containing a wt branch site or one or two of disrupted (A-to-G mutation) branch sites as diagramed in Fig. 4A and examined the mapped branch sites in regulation of HPV18 E6E7 RNA splicing and production of E6 and E7 proteins in HEK293T and U2OS cells. To correlate E6E7 splicing with the production of E6 and E7 proteins, we also fused a Flag-tag in frame to E6 at its N-terminus and to E7 at its C-terminus, respectively, in the corresponding expression vectors (Fig. 4A). Total RNA and total proteins were respectively extracted from the cells after 24 h of transfection and were compared for splicing efficiency of the spliced E6E7 mRNA isoforms by RT-PCR (Fig. 4) and production of E6 and E7 proteins by Western blotting (Fig. 5). As shown in Fig. 4B, the splicing patterns of wt and mt-1 E6E7 pre-mRNAs in HEK293T and U2OS cells were similar, with high production of the spliced E6*I mRNA (97%–99% in U2OS cells) and minimal level of unspliced E6E7 RNA (5%–11% in HEK293T cells). Comparisons between mt-2 and wt transfected cells showed that the proportion of E6*I mRNA production from mt-2 containing an A-to-G mutation at nt 388 reduced to ~ 39% in HEK293T cells and to ~ 62% in U2OS cells, accompanied by a corresponding increase of the unspliced E6E7 RNA (compare lanes 7 to 3 and 17 to 13). Moreover, we observed a novel spliced mRNA (233^636) from a cryptic splicing acceptor site at nt 636 in the mt-2 transfected U2OS cells (lane 17), but not in the wt transfected cells (Fig. 4B–4D). We didn’t see any 233^636 splicing in mt-2 transfected HEK293T cells (lane 7). However, this aberrant 233^636 splicing happened in both HEK293T and U2OS cells transfected with the mt-3 vector which contains the A-to-G mutation both at nt 384 and nt 388 (lanes 9 and l9). In this case, the mt-3 exhibited no E6*I RNA splicing and remained 97% or 79% of E6E7 RNA with no splicing, respectively, in both HEK293T (lane 9) and U2OS (lane 19) cells. These data clearly show that HPV18 E6*I RNA splicing is mediated by two alternative branch sites in the cells as shown in *in vitro* splicing and the 3′ ss-proximal branch site at nt 388 is more potent than its distal branch site at nt 384 in regulation of E6*I RNA splicing.

It has been well established that the E6*I RNA serves as an E7 mRNA and the E6 intron splicing disrupts integrity of the E6 ORF and prevents production of full-length E6 protein (Zheng *et al.*^2004^; Tang *et al.*^2006^; Ajiro *et al.*^2012^). Subsequently, effect of the mapped branch sites at nt 384 and 388 on production of HPV18 E6 and E7 proteins were examined by Western blotting using the total protein extracts derived from individual vector-transfected HEK293T or U2OS cells as described above. As shown in Fig. 5, we found that the increased production of viral E6 protein both in HEK293T and U2OS cells was accompanied by the decreased expression of viral E7 protein level. This inversed expression of E6 and E7 proteins from the same bicistronic E6E7 RNA was in a linear correlation with the increased escaping of E6 intron splicing from the bicistronic E6E7 RNA transcripts (Fig. 4). As expected, the majority (> 95%) of E6E7 pre-mRNA transcripts with wt branch sites were efficiently spliced as an E6*I RNA (Fig. 4B, lanes 3 and 13), leading to predominantly produce E7 protein both in HEK293T and U2OS cells (Fig. 5A–5C, lanes 3). Minimal amount of E6 protein expressed from the same wt vector was encoded from the residual unspliced E6E7 RNA (Fig. 5A, 5C, lanes 3). Disruption of either one of mapped branch sites (mt-1 or mt-2) slowed down the efficiency of E6*I splicing (Fig. 4B, lanes 5, 7, 15 and 17) and increased E6, but decreased E7 protein production, in particular for the mt-2 with A-to-G mutation in the mapped branch site at nt 388, a 3′ss-proximal branch site (Fig. 5A–5C, compare lanes 4-5 to lanes 3 in both types of cells). Furthermore, disruption of both mapped branch sites was found to block E6E7 RNA splicing (Fig. 4B, lanes 9 and 19), resulting in production of plentiful E6 protein with little detectable E7 from this mt-3 vector (Fig. 5A–5C, compare lanes 6 with lanes 3-5 in both types of cells). Altogether, these data indicate the mapped branch sites are essential for E6E7 RNA splicing to regulate the production of viral E6 and E7 proteins. In this regard, the 3′ss-proximal branch site at nt 388 is more potent than its distal branch site at nt 384 in regulation of E6 and E7 protein production.

## Discussion

In HR-HPV, splicing of the E6 intron from a bicistronic E6E7 pre-mRNA is a crucial step to control expression of E6 and E7 oncogenes (Zheng *et al.*^2004^). An efficient RNA splicing depends on multiple RNA cis-elements including a functional BPS (a mammalian consensus heptamer YNYURAC) in each intron and cellular splicing factors (Zheng 2004; Lee and Rio 2015; Shi 2017). We had previously mapped the branch site of HPV16 E6 intron splicing to an adenosine at nt 385 (Ajiro *et al.*^2012^). In this study, we identified that HPV18 E6 intron utilizes two alternative branch sites, one at nt 384 and the other preferential one at nt 388, for its RNA splicing and expression of viral E6 and E7.

Although both HPV16 and HPV18 E6 introns initiate its first step splicing by using an adenosine nucleotide as a branch site at the sixth position of the mapped heptamer BPS, the sequence composition of the mapped BPS in the HPV16 E6 intron is AACAAAC, whereas in the HPV18 E6 intron is AACUAAC, a duplicate sequence from nt 379 to 385 and from nt 383 to 389. Thus, the mapped BPS in the HPV18 E6 intron differs from that of the HPV16 intron only at the fourth nucleotide position, U for HPV18 and A for HPV16, leading the mapped HPV18 E6 BPS a better CV score (77%) over the HPV16 E6 BPS (62%) (Ajiro *et al.*^2012^) to the consensus sequence YNYURAC of a mammalian BPS. Despite that multiple adenosines are present in each mapped BPS, our point mutation studies indicate that only the adenosine at the sixth position in the mapped BPS was used as a branch site during E6 RNA splicing. These data clearly show how accurately the cellular splicing machinery executes U2 snRNP recognition of each mapped BPS in HPV18 E6 splicing. By sliding only four nucleotides, U2 could simply switch to another alternative BPS to regulate the efficiency of E6 intron splicing.

Successful mapping of the two alternative BPSs in control of HPV18 E6 intron splicing also lead us to define the length and composition of a PPT between the mapped BPS and the nt 416 3′ss. Depending on which branch site is selected for HPV18 E6 intron splicing, the PPT at this 3′ss could be either in size of 26 nts if the nt 388 branch site is used or 30 nts if the nt 384 branch site is chosen. In either case, the 26-nt PPT is interrupted by its 12 purines (9 As and 3 Gs) and the 30-nt PPT is interrupted by its 14 purines (11 As and 3 Gs) upstream of the AG dinucleotides at the 3′ss. PPT composition of the mixed pyrimidines and purines, in particular cytosine and adenosine, makes the PPT being suboptimal in interaction with U2AF and other related splicing factors necessary for U2 recruitment (Berglund *et al.*^1998^; Sickmier *et al.*^2006^; Tavanez *et al.*^2012^; Agrawal *et al.*^2016^; Sutandy *et al.*^2018^) and affects efficiency of RNA splicing (Zamore *et al.*^1992^; Sohail and Xie 2015). Since the 26-nt PPT has fewer adenosines than the 30-nt PPT and thereby is a relatively stronger PPT over the 30-nt PPT, this feature of the 26-nt PPT may explain why the 3′ ss-proximal branch site at nt 388 is preferentially used for HPV18 E6*I splicing. Nevertheless, the importance of the nt 388 branch site in the E6*I splicing was also revealed by disruption of the proximal branch site to activate the usage of a cryptic acceptor site at nt 636 and lead to aberrant 233^636 splicing, which was not previously noted.

It has been well-established that the E6 intron splicing from a bicistronic E6E7 pre-mRNA creates a premature stop codon to increase intercistronic space for efficient translation termination-reinitiation and thus is crucial for generating E6*I RNA to translate E7 protein, while the E6 intron retention is necessary for keeping the E6 ORF integrity for production of E6 protein (Zheng and Baker 2006; Tang *et al.*^2006^; Zheng 2010; Ajiro and Zheng 2014). By transfection of HEK293T and U2OS cells with individual branch site-mutated constructs, we showed that the branch site at nt 388 was more potent than the branch site at nt 384 not only for the E6*I splicing but also for the production of E7 proteins. We observed that efficient production of the spliced E6*I mRNA led to efficient production of E7 proteins, but less amount of E6 protein. The reverse was true for more retention of the E6 intron due to less E6*I splicing. Despite existence of the branch site selection bias, we are assuming that both branch sites are useful for HPV18 infection. By alternative selection of the two mapped branch sites for splicing of bicistronic E6E7 pre-mRNA, HPV18 is capable to justify its production ratio of the two oncogenic, multifunctional E6 and E7 proteins along with its progression of productive infection, cell immortalization and transformation (Fig. 5D). This hypothesis is in consistent with the recent observation that the most human introns are recognized by multiple and tissue-specific BPS (Pineda and Bradley 2018). However, further studies are needed to understand what host splicing factor(s) contributes to selection of the proximal over the distal branch site for E6*I splicing despite that the expression of host splicing factors remains changing from undifferentiated keratinocytes in the basal layer to highly differentiated keratinocytes in the spinal/granular layers (Fay *et al.*^2009^; Mole *et al.*^2009^; Ajiro *et al.*2016a).

## Electronic supplementary material

Below is the link to the electronic supplementary material.

Supplementary material 1 (PDF 332 kb)
