# Supplementary material for: HPV18 Utilizes Two Alternative Branch Sites for E6*I Splicing to Produce E7 Protein
Source: Virol Sin. 2019 Apr 3;34(2):211–21. doi: 10.1007/s12250-019-00098-0 (PMC6513837; doi:10.1007/s12250-019-00098-0)
Supplement: Supplementary file 1 — Supplementary material 1 (PDF 332 kb) [file 12250_2019_98_MOESM1_ESM.pdf]

VIROLOGICA SINICA

Electronic Supplementary Material

HPV18 Utilizes Two Alternative Branch Sites for E6*I Splicing to Produce E7 Protein

Ayslan Castro Brant[1,2,3], Vladimir Majerciak[1], Miguel Angelo Martins Moreira[3], Zhi-Ming Zheng[1]✉

1. Tumor Virus RNA Biology Section, RNA Biology Laboratory, Center for Cancer Research, National Cancer Institute, NIH, Frederick, Maryland 21702, United States of America.
2. Genetics Post-Graduation Program, Rio de Janeiro Federal University, Rio de Janeiro, Brazil.
3. Genetics Program, Nacional Cancer Institute, INCA, Rio de Janeiro 20231-050, Brazil.

Supporting information to DOI: 10.1007/s12250-019-00098-0

Table S1: Primers sequence used in the study.

Primer	Sequence (5′-3′)	Genome	5′NT	3′NT	Strand	Comments
oAYS8	CTAGAATTAGAGAATTAAGAC	HPV18	322	342	Plus	Lariat RT-PCR (F2)
oAYS12	GGGGTATACTGTCTCTATACACCACA	HPV18	288	263	Minus	Lariat RT-PCR (R)
oAYS13	GCTGCATGCCATAAATGTATAGATTT	HPV18	291	316	Plus	Lariat RT-PCR (F1)
oAYS20	ATAACCCAGTGTTAGCTAGTTTTTCCAATGTGTCT	HPV18	399	365	Minus	BPS A384G mutation
oAYS21	AGCTAACACTGGGTTATACAATTTATTAATAAGGT	HPV18	383	417	Plus	BPS A384G mutation
oAYS22	ATAACCCAGTGCTAGCTAGTTTTTCCAATGTGTCT	HPV18	399	365	Minus	BPS A384G and A388G mutation
oAYS23	AGCTAGCACTGGGTTATACAATTTATTAATAAGGT	HPV18	383	417	Plus	BPS A384G and A388G mutation
oAYS41	ATAACCCAGTGCTAGTTAGTTTTTCCAATGTGTCT	HPV18	399	365	Minus	BPS A388G mutation
oAYS42	AACTAGCACTGGGTTATACAATTTATTAATAAGGT	HPV18	383	417	Plus	BPS A388G mutation
oAYS40	ACTAAGCTT/CTATGGCGCGCTTTGAGGATC	HPV18	103	123	Plus	Overlapping PCR
oMA77	CTGAGCGGCCGC/AAACCAGCCGTTACAACCCG	HPV18	967	948	Minus	Overlapping PCR
oSB70	TAATACGACTCACTATAGGG/CCAACACGGCGACCCTAC	HPV18	123	140	Plus	T7 promoter+18E6
oST247	GTACTCACCCC/AGCTATGTTGTGAAATCG	HPV18	500	480	Minus	18E7+U1-binding site
oZMZ252	ATCCAACACGGCGACCCTAC	HPV18	121	140	Plus	RT-PCR (HPV18E6E7)
oZMZ253	CTGGAATGCTCGAAGGTC	HPV18	850	833	Minus	RT-PCR (HPV18E6E7)
oZMZ437	ATCGGAATTCACC/ATGGACTACAAAGACGATGACGAC/A TGGCGCGCTTTGAGGATC	HPV18	105	123	Plus	EcoRI+FLAG+18E6
oZMZ433	CACTGAGGTAC/CTGCTGGGATGCACACCAC	HPV18	904	886	Minus	18E7+KpnI
oZMZ269	GTCATCAATGGAAATCCCATCACC	hg19: chr12	6645922	6645945	Plus	RT-PCR (GAPDH)
oZMZ270	TGAGTCCTTCCACGATACCAAA	hg19: chr12	6646554	6646533	Minus	RT-PCR (GAPDH)

Underline – Mutated bases.
